# Formation of side discharges in dielectric barrier discharge

**DOI:** 10.1038/s41598-017-08470-4

**Published:** 2017-08-21

**Authors:** Weili Fan, Zhengming Sheng, Lifang Dong, Fucheng Liu, Xiaoxia Zhong, Yiqian Cui, Fang Hao, Tian Du

**Affiliations:** 1 Key Laboratory for Laser Plasmas (MoE) and School of Physics and Astronomy, Shanghai Jiao Tong University, Shanghai, 200240 China; 2 Collaborative Innovation Center of IFSA (CICIFSA), Shanghai Jiao Tong University, Shanghai, 200240 China; 3 College of Physics Science and Technology, Hebei University, Baoding, 071002 China; 4 SUPA, Department of Physics, University of Strathclyde, Glasgow, G4 0NG UK

## Abstract

Pattern formation and self-organization are fascinating phenomena found widely in nature and in laboratory environment such as dielectric barrier discharge (DBD). Significant efforts have been made to explain the dynamic pattern formation. In DBD, the formation of side discharges is generally supposed to be a key factor responsible for diversity and spatial-temporal symmetry breaking of pattern formation. However, it is still not clear how such discharges are induced. Here, we present the observations of side discharges in a filamentary dielectric barrier discharge from both numerical simulations and experiments. Two-dimensional particle-in-cell simulations with Monte Carlo collisions included have revealed formation dynamics of side discharges, suggesting that transverse plasma diffusion and ion induced secondary electron emission play critical roles. Moreover, a novel honeycomb superlattice pattern is observed in experiment, where the side discharges associated with honeycomb superlattice are verified by utilizing a high speed camera. Experimental observations and numerical simulation are in good agreement.

## Introduction

From zebra stripes to a honeycomb lattice, nature features various breathtaking patterns, which have aroused a lot of fascination and puzzle throughout human history^1, 2^. For the past decades, extensive researches on self-organized pattern formation have been carried out in different laboratory systems^3^, such as the reaction-diffusion system, the Faraday system, the nonlinear optical system and the hydrodynamics. Recently, increasing attention has been paid to the dielectric barrier discharge system (DBD), which is capable of producing the most varieties of patterns with simple experimental setup^4–14^. These plasma patterns generally exhibit high spatial-temporal symmetries at the macroscopic level, such as the hexagonal or square Bravais lattices in space and the harmonic or subharmonic symmetry in time. However, from the microscopic view, the filaments are always characterized by complex dynamics and interactions. These intriguing phenomena pose deep questions on the underlying physics of DBDs.

The formation of side discharges is generally supposed to be a key factor responsible for diversity and rich dynamical behaviors of DBD patterns. It refers to the discharges generated during the same half-cycle in the vicinity of an isolated filament beyond the inhibition zone^8^. It gives rise to spatial-temporal symmetry breaking of pattern formation and can explain many aspects of filament interactions. To date, most studies on side discharges are concentrated on experimental observations. Gurevich *et al*. obtained a concentric-ring pattern, which is a superposition of two complementary forms of rings. These two forms have a harmonic temporal behavior, in which one form is the side discharges of the other^9^. Walhout *et al*. categorized the plasma patterns into three different types. In their ‘Type B’ pattern, an additional discharge is ignited in the spacing between the primary isolated filaments^10, 11^, which can be considered as the side discharges. Our group previously observed a square pattern, which consists of two subharmonic square sublattices. One sublattice occurs outside the regions delimited by the other^12^. However, in spite of these experimental measurements, the involved physics associate with side discharges is still not completely clear. Several fluid simulations on formation of side discharges have been performed^8, 13^, with the assumption of the Maxwell-Boltzmann velocity distribution of the plasma particles. It is supposed that the initiation of side discharge is associated with the charge spreading along the dielectric surface. To our knowledge, there is no kinetic simulation studies reported on this issue. With great advantages of kinetic fidelity, the kinetic simulation may reveal new physics important for understanding the complicated behaviors of the discharge^15–20^.

In this paper, we investigate the formation of side discharges in DBD both in experiment and numerical simulation. We present the first kinetic simulation on side discharges by using two-dimensional simulations with Monte Carlo collisions included (PIC-MCC). In experiment, a novel honeycomb superlattice pattern is observed in a DBD system with two water electrodes. The existence of side discharges in the honeycomb superlattice is verified by using the high speed camera. The correspondence of experimental and numerical findings is strikingly good.

## Models and Methods

**Figure 1:**
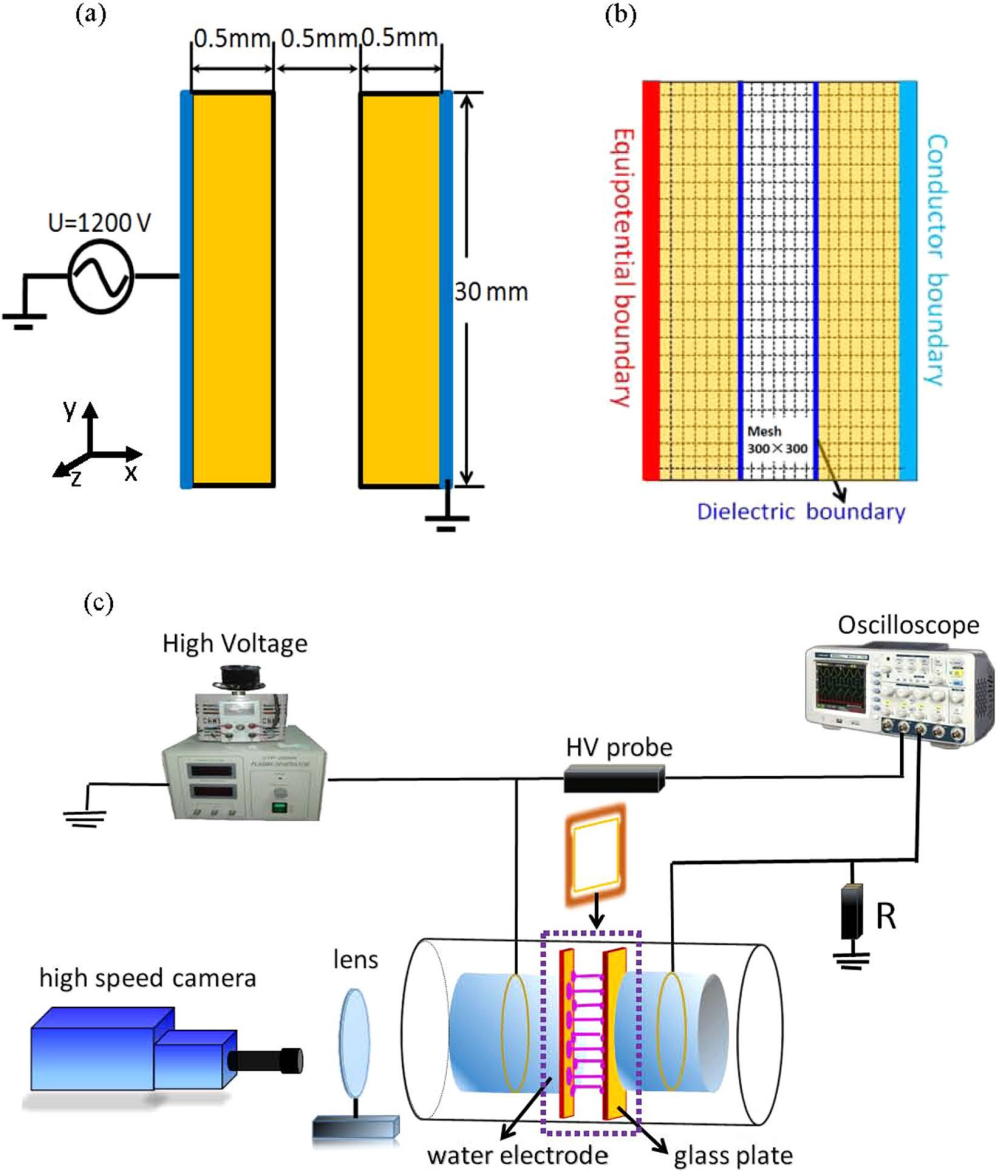
Schematic diagrams of the dielectric barrier discharge used in simulation (**a**,**b**) and in experiment (**c**). The configuration within the dashed regions in (**c**) is similar to our simulation model.

The schematic diagrams of the simulation model and the experimental setup are shown in Fig. 1. The simulation model consists of two parallel plate electrodes covered with 0.5 mm-thick dielectric layers (the permittivity *ε* = 7.6). A thin MgO film, whose ion-induced secondary electron emission coefficient (SEEC) is set as 0.4, is painted on the surface of the dielectric layer. The discharge is sustained in helium gas at the pressure of 200 Torr, and the gas gap is 0.5 mm. Thus the discharge is operated in the glow discharge regime with *pd* ≤ 10 Torr cm. A sinusoidal ac voltage with the frequency *f* = 200 kHz and magnitude *U* = 1200 V is applied to the left electrode, and the right electrode is grounded. The simulation starts from *U* = 0 V at *t*_0_ and then the voltage increases following a sinusoidal curve.

The discharge is simulated using the object-oriented 2d3v PIC-MCC code XOOPIC^21^. At the left boundary x = 0, an equipotential boundary is specified, whose potential is given by the applied voltage. The right boundary is a perfect conductor that is grounded. These two kinds of boundaries are given as Dirichlet boundary conditions. At the interfaces between plasma and dielectric layers, as indicated by dark blue lines in Fig. 1(b), dielectric boundary conditions are defined. Charges can accumulate on the dielectrics as particles hit the surface. It is assumed that the positions of surface charges are fixed once they touch the dielectric layer, which can neither move along the dielectric surface nor draw back into the gas gap in subsequent discharges. The computational grid is defined to be 300 × 300 cells, in which the geometric spacing is chosen to be uniform in both directions. This grid corresponds to 1.5 mm horizontally and 30 mm vertically for the modeled device. The time step in simulation is set as 10^−13^ s. The simulations track electrons and He^+^ ions while the distribution of background neutrals is assumed to be time independent and uniform in space. In the procedure of Monte Carlo collisions, elastic, excitation, and ionization collisions between electrons and neutrals are accounted for. For ions, the elastic scattering and charge exchange collisions are included. The cross-sectional data set used in this paper is the same with ref. 22. The initial densities of both electrons and ions are distributed uniformly at 10^15^ m^−3^. This is a basic model to give a qualitative explanation on the dynamical aspects of DBD filaments. Although the detailed results may be sensitive to parameters such as the presence of dimer ions, the dimer excited states, the fundamental discharging process and trends discussed in this paper are very general. The results are well consistent with the experimental measurements as well as the fluid models^8, 13^.

In experiment, a dielectric barrier discharge with two water electrodes is utilized, as shown in Fig. 1(c). The electrodes are two cylindrical containers sealed with glass plates and filled with water. A metallic ring is immersed in each container and connected to a sinusoidal ac power supply. A square glass frame is clamped between the two parallel glass plates, serving as a lateral boundary. The whole cell is placed in a big chamber, where the gas pressures can be changed. An intensified charge-coupled device camera (HSFC pro) is applied to record frames from the end view of the electrodes. A detailed description of the experimental setup can be found in refs 23 and 24.

## Results and Discussion

### Simulation results

**Figure 2:**
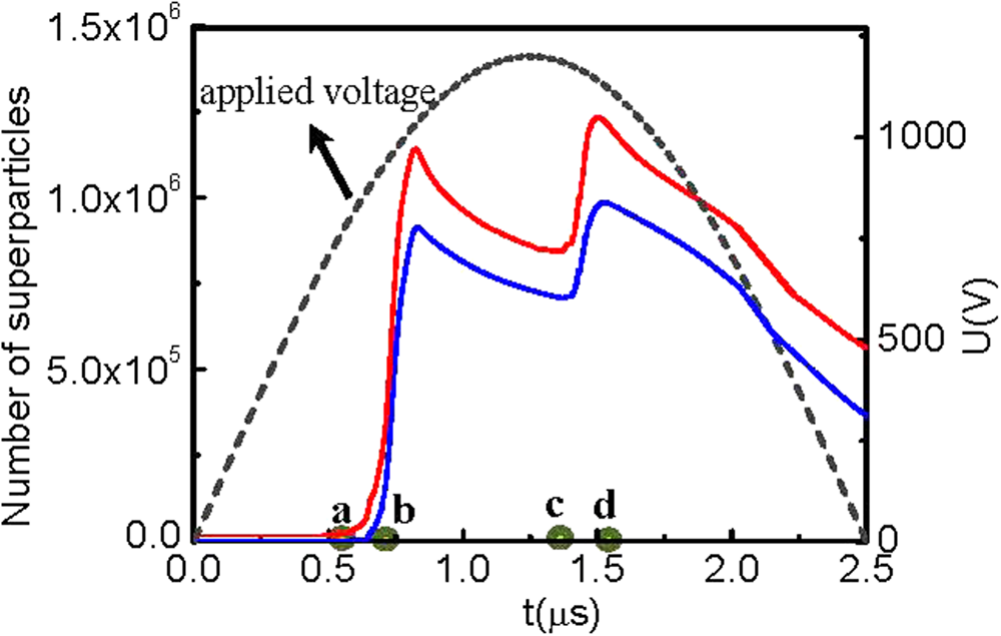
Time trace of the number of superparticles in the gas gap, where the red line is for He^+^ and the blue line is for electrons. One superparticle corresponds to 10^7^ real particles. The green circles (**a** to **d**) indicate the times discussed later in Figs 3 to 4. The times are t = 0.62 μs, 0.72 μs, 1.34 μs, 1.51 μs, respectively.

Figure 2 shows the time trace of the number of superparticles in the gas gap. The observation of two narrow consecutive pulses means that the ignition of discharge takes place two times during one half period of the supply voltage. The first pulse corresponds to the discharge of primary isolated filaments, while the second one corresponds to the side discharges.

**Figure 3:**
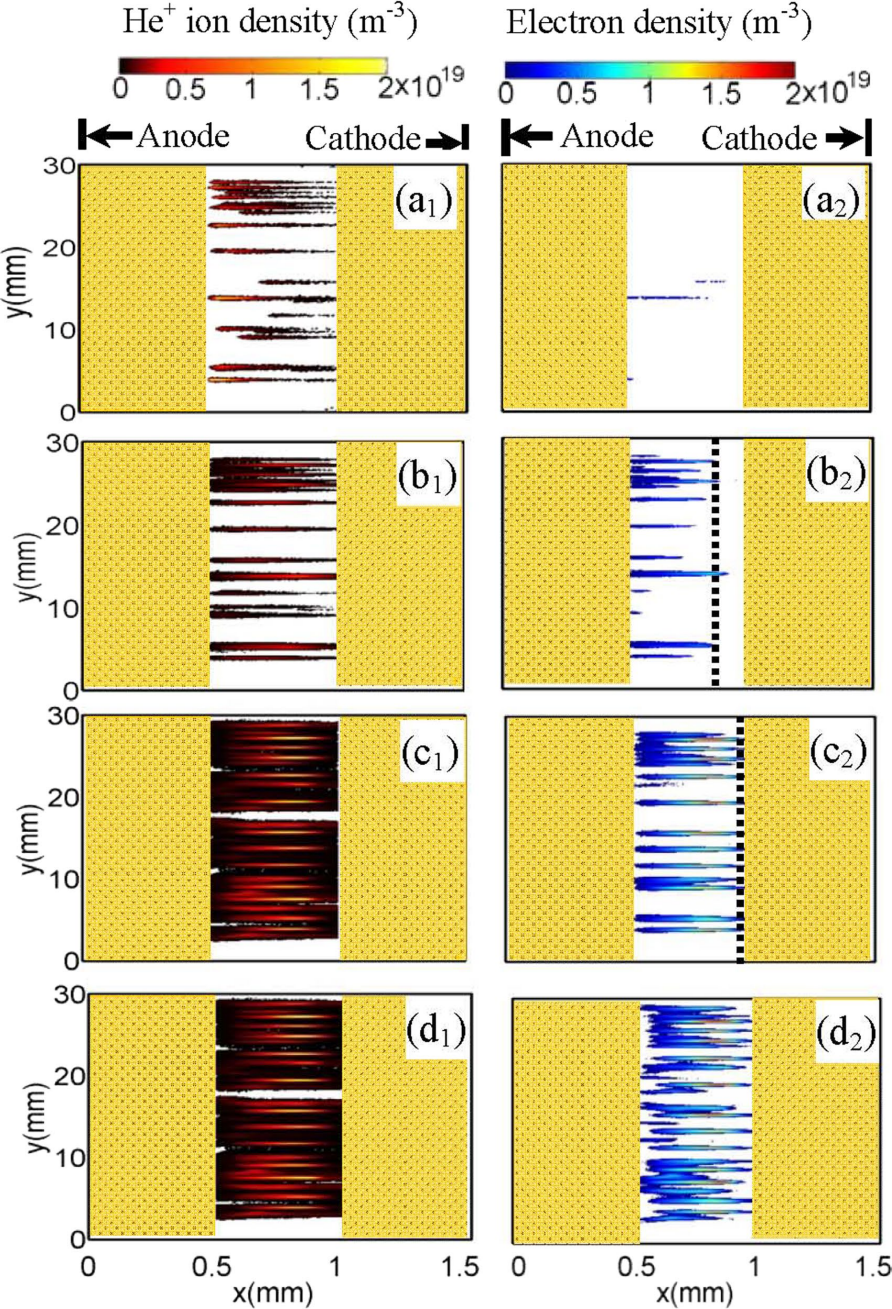
Time evolution of filamentary discharges in DBD. The axes (Anode and Cathode) denote the surfaces of metal electrodes. The values of densities displayed in (**a**_**1**_) and (**a**_**2**_) have been multiplied by a factor of 16. The dashed lines in (**b**_**2**_) and (**c**_**2**_) show the edges of the transient plasma sheaths.

**Figure 4:**
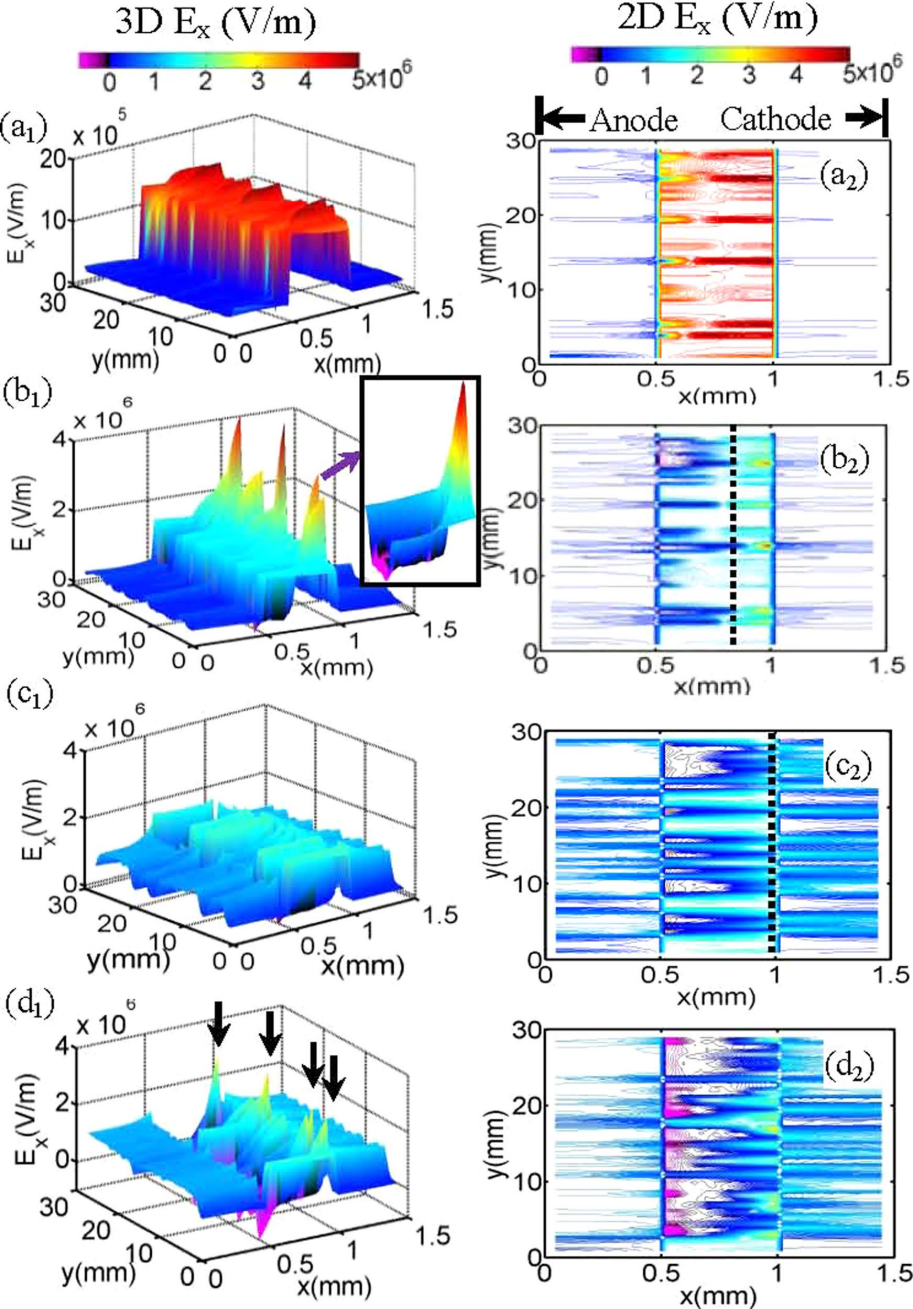
Evolution of E_x_ as the discharge proceeds. The first column shows the 3D E_x_ profiles while the second column presents the corresponding contour graphics. The E_x_ profile at y ∈ [13.4 mm, 14.4 mm] is magnified in the inset nearby. The dashed lines in (**b**_**2**_) and (**c**_**2**_) show the edges of the transient plasma sheaths. The black arrows in (**d**_**1**_) indicate the E_x_ peaks where the side discharges occur.

The evolution of the discharge is presented in Fig. 3. Firstly, the primary isolated filaments are initiated, as shown in Fig. 3(a–c). It can be divided into three different phases: the Townsend phase (Fig. 3(a)), the space-charge dominated phase (Fig. 3(b)), and the decay phase (Fig. 3(c)), as the discharge proceeds. It is similar to the normal filamentary discharges as demonstrated in our previous studies^19, 20^. At the Townsend phase, the discharge is just ignited and multiple avalanches develop in a space-electrons free field. The electrons produced in avalanches will quickly accumulate on the left dielectric sheet (Fig. 3(a_2_)), while the ions move slowly towards the right dielectric. These ions gain sufficient energy with the increase of the sinusoidal voltage swing, and some energetic ones induce the secondary electron emission when hitting the surface of the right dielectric layer. When sufficient electrons have been deposited on the left dielectric layer, they will build up an opposite field near the dielectric layer (as shown later in Fig. 4(b_1_)) to prevent further accumulation of the subsequent electrons produced in avalanches. Consequently, the electrons begin to accumulate in the gas gap, and the discharge comes into the space-charge dominated phase (Fig. 3(b)). The discharge of this phase develops very intensely, which is characterized by multiplication of avalanches occurring in plasma sheaths. Here we should point out that we define the boundary where the distribution of the electrons and ions separates as the edge of the plasma sheath, where a remarkable distortion of electric field will take place (as shown later in Fig. 4). Each filament has its own plasma sheath, which depends on its developing level. For simplicity, we roughly use one dashed line to denote the edges of the plasma sheaths of all filaments. The plasma particles proliferate as the discharge proceeds, and the plasma sheath gradually moves to the right dielectric layer. When the edge of the sheath reaches the right dielectric layer (as shown in Fig. 3(c_2_)), these filaments are extinguished. Interestingly, the extinction of these primary filaments does not suggest the ending of the discharge. The side discharges start to appear with further increase of the applied voltage swing, as shown in Fig. 3(d_2_). They preferentially form on the two sides of the primary filaments and generally exhibit as thin and short filaments, which emerge only in the vicinity of the dielectric layer covering the anode. Moreover, the side discharges are generally low current discharges that produce ion and electron densities lower by one order of magnitude than the primary filaments.

To further clarify the mechanism of the discharges, Fig. 4 plots snapshots of the total electric field along x direction (E_x_) and their corresponding contour lines. We define the electric field along + x direction as positive. Obviously, when the discharge is in the Townsend phase (Fig. 4(a)), the E_x_ profile in the gas gap x ∈ [0.5 mm, 1 mm] displays some perturbations superimposed over the uniform background. The field E_x_ are nearly not influenced by the space charges. When the discharges transit to the space-charge dominated avalanche phase (Fig. 4(b)), multiple trenches with steep peaks near the right dielectric layer emerge. The trenches correspond to the bulk plasma regions with E_x_ = 0, while the sharp peaks correspond to the plasma sheaths where a strong electric field directed to the cathode is produced. By accelerating the charged particles, the E_x_ peaks indicate the positions of the discharging filaments. The transient sheath takes place over a shorter and shorter length between the cathode and the bulk plasma with the development of the discharge (Fig. 4(b_2_,c_2_)). Meanwhile, the E_x_ peaks in plasma sheaths gradually declines. When the sheaths reach the right dielectric layer, the primary discharges expire and all of the peaks disappear (Fig. 4(c)). However, with initiation of the side discharges, multiple E_x_ peaks are activated again, which emerge on the two sides of the primary isolated filaments (Fig. 4(d)). Here the magnitude of E_x_ peaks is obviously reduced compared with that of primary discharges, leading to weak intensity of the side discharge.

**Figure 5:**
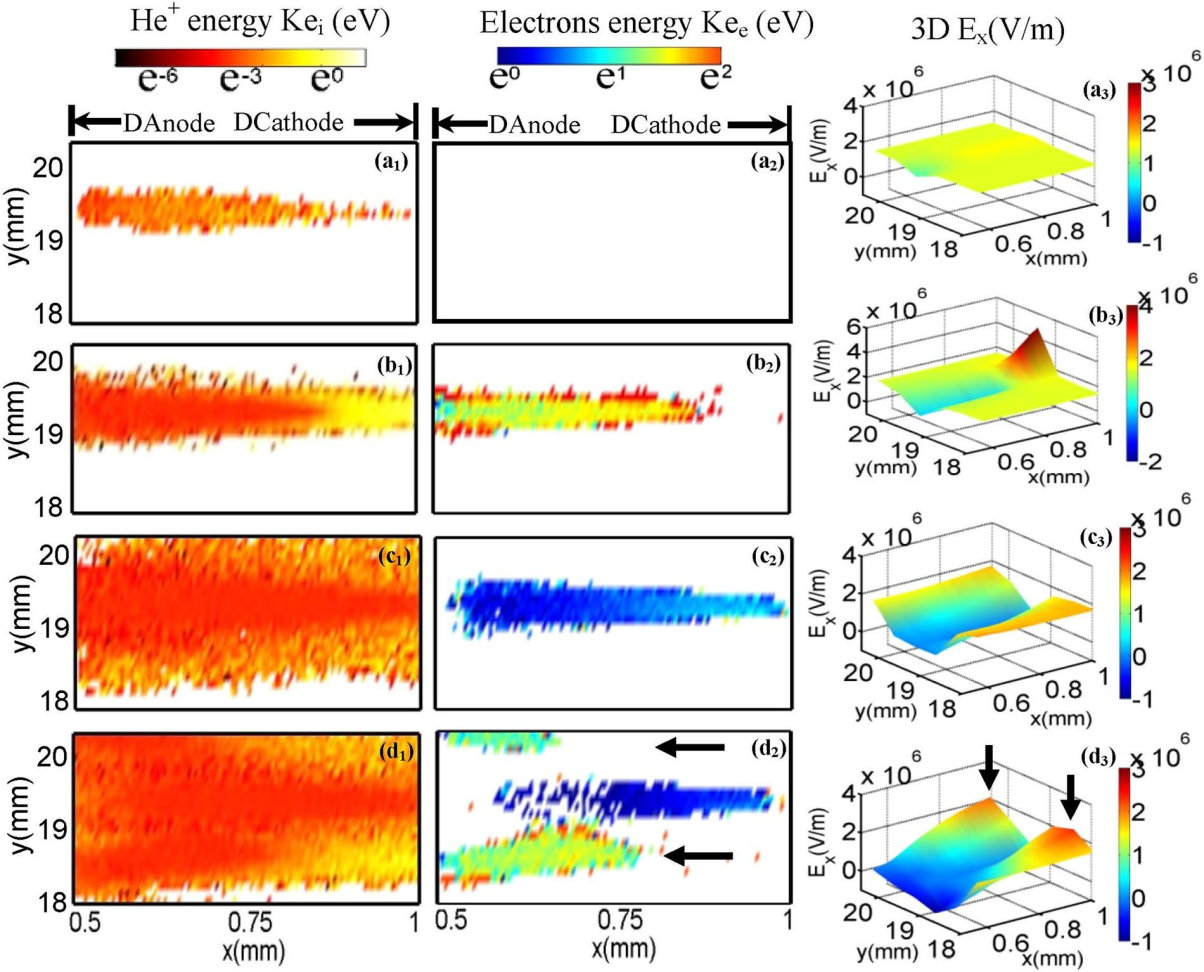
Development of the side discharges around a single filament. The times are t = 0.62 μs, 0.74 μs, 1.34 μs, 1.51 μs, respectively. The left and middle columns denote the kinetic energy distributions of He^+^ ions and electrons in the gas gap. To cover a wide range, logarithm coordinates are adopted for the colorbar label. The right column illustrates the corresponding 3D E_x_ distributions. The filament corresponds to the one y ∈ [18 mm, 20.4 mm] as shown in Fig. 3. The black arrows in (**d**_**2**_) indicate the positions where side discharges occur.

We now examine the kinetic energy distribution of the charged particles in the gas gap, which provides a good explanation on formation of the side discharges. For the primary discharges as shown in Fig. 5(a–c), the kinetic energy of the electrons is mainly in the order of e^1^~e^2^ eV when the filament is in the space-charge dominated phase (Fig. 5(b_2_)). It decreases to e^0^~e^1^ eV as soon as the discharge has terminated (Fig. 5(c_2_)). The ignition of side discharges can be easily identified in Fig. 5(d_2_), in which appreciable high energy electrons emerge around the initial isolate filaments. The plasma growth is activated again in the regions indicated by the black arrows.

A joint action of both the transverse plasma diffusion and the ion induced secondary electron emission beyond the inhibition zone leads to initiation of the side discharges. As shown in Fig. 5(c), we can find that the ion channels are much wider than that of electrons. This is attributed to the fact that it takes longer time for heavier ions to go across the gas gap, giving rise to enough time to diffuse transversely. Besides, the kinetic energy of the ions at the two margins of the channel is significantly larger than that in the middle (Fig. 5(c_1_)), which corresponds to the inhibition region where E_x_ ≈ 0. These ions, which are away from the inhibition region, are greatly accelerated by the applied voltage. They strike on the right dielectric layers as the seed charges to induce secondary electron emission and therefore activate the side discharges in the space that was free of discharge in the primary one. Moreover, we can also find that the side discharge undergoes a transition from the Townsend phase to the glow phase as the discharge proceeds. As shown in Fig. 5(c_3_), when the side discharge just starts, the electric field away from the inhibition region is uniform without distortions. This corresponds to the Townsend discharge, in which the electric field distribution is not perturbed by space charges. The emergence of E_x_ peaks as shown in Fig. 5(d_3_) indicates the transition from the Townsend phase to the glow phase. The electric field now is significantly distorted due to the accumulation of space charges. Our results are slightly different with that of fluid simulations^13^, in which the side discharge is associated with the Townsend discharge.

**Figure 6:**
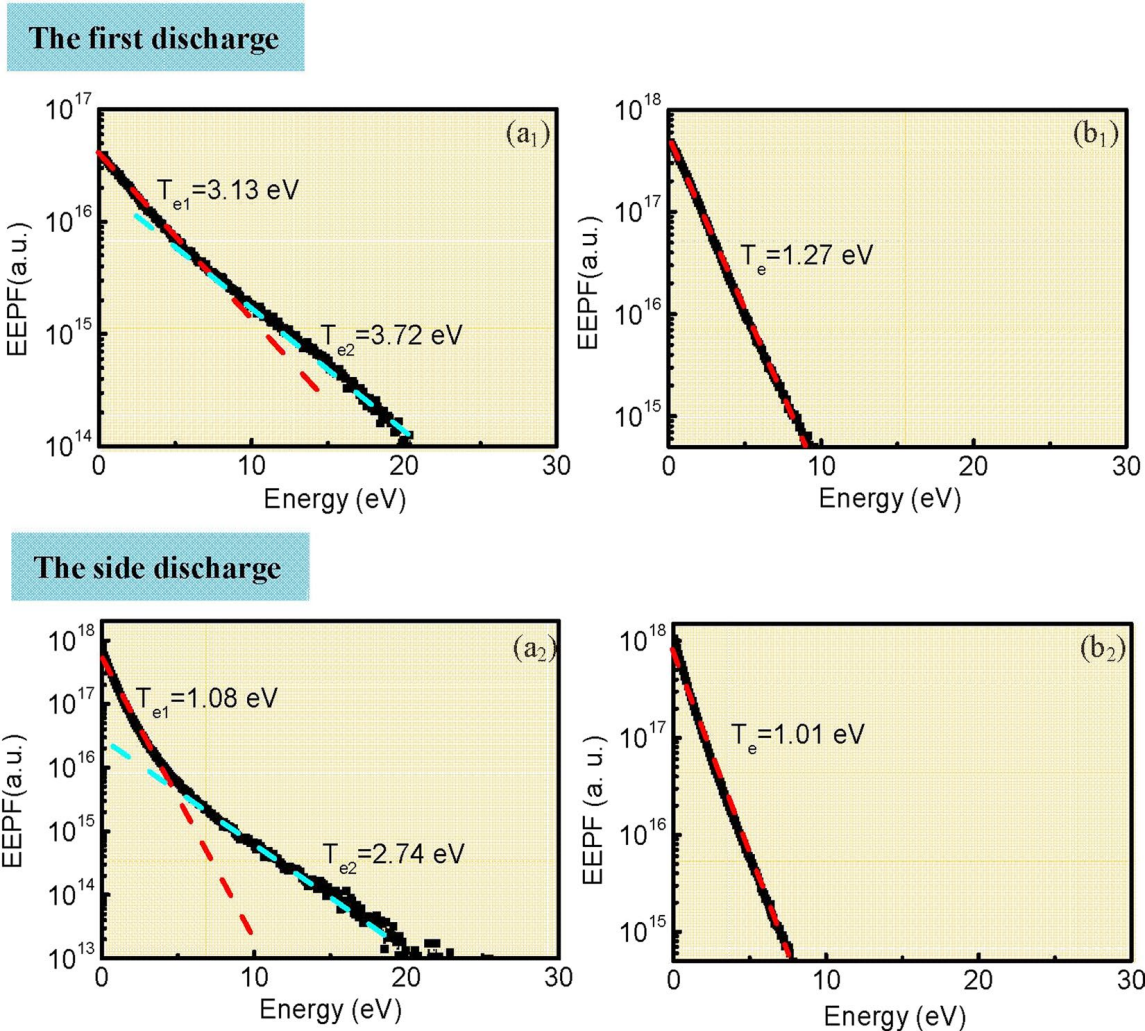
Evolution of EEPFs during the first discharge (**a**_**1**_,**b**_**1**_) and side discharge (**a**_**2**_,**b**_**2**_). The time instants are (**a**_**1**_) t = 0.72 μs, (**b**_**1**_) t = 1.34 μs, (**a**_**2**_) t = 1.51 μs, (**b**_**2**_) t = 1.97 μs. In (**a**_**1**_) and (**a**_**2**_), the discharge is in the space-charge dominated avalanche phase, while in (**b**_**1**_) and (**b**_**2**_), the discharges have expired. T_e1_ and T_e2_ denote the low and high electron temperatures, respectively.

In accordance with the kinetic energy distribution of the charged particles, Fig. 6 presents the electron energy probability functions (EEPFs) and the electron temperatures at different moments. It indicates that (1) no matter in the primary discharge or in the side discharge, the EEPF shows a bi-Maxwellian distribution during the discharge (Fig. 6(a_1_) and (a_2_)), and it evolves into the Maxwellian distribution when the discharges expire (Fig. 6(b_1_) and (b_2_)). Besides, the electron temperature decreases as each discharge proceeds. (2) Compared with the primary discharge, the electron temperature of side discharges is much reduced, *i*.*e*. the side discharge is weak. This is well consistent with the decrease of E_x_ peaks as displayed in Fig. 4(d). (3) The plasma exhibits more non-equilibrium during the side discharges, which is characterized by a larger difference between T_e1_ and T_e2_. This is reasonable since appreciable low-energy electrons that produced in primary discharges survive, which can be clearly seen from Fig. 5(d_2_).

## Discussion on the Methodology

We have adopted particle-in-cell simulation with Monte Carlo collisions included (PIC-MCC)^19, 20^ in this work. Comparing with the fluid simulation, simulations with PIC-MCC enable one to model the formation of microstructures related to kinetic features during the discharge processes, such as filamentary formation, nonthermal distributions of particles, etc. For example, generally it is believed that DBD in the glow regime is nearly homogeneous over the electrode in the first one or two pulses of discharge in fluid simulations. However, here the formation of filamentary microstructures is a natural result of Monte Carlo calculation involved in a few processes such as free electron emission from cathode and impact ionization by high energy particles. Since energetic particles are relatively few and are not all ideally distributed uniformly, there are naturally fluctuations (or local non-uniformity) in their spatial distributions of charges, even if they are statistically uniform. Such filamentary microstructures have also been observed in ref. 25. However, despite the deviations for the initial discharging process between the kinetic and fluid models, the fundamental mechanism of side discharges found from our particle simulation is consistent with the fluid models^8^. Moreover, to our best knowledge, there is no experimental data reported on related measurements with such high space and time resolutions as described in our paper. In many cases, the discharge looks uniform but is actually composed of many random filaments at a micro level. It is worthwhile to point out that the establishment of self-organized patterns in DBD generally takes several or many cycles. It is a complicated process resulting from a nonlinear interaction between the plasma filaments and surface charges. Here we just discuss part of the physics related to the formation of side discharge, rather than complete mechanisms of pattern formation.

### Comparisons of numerical and experimental results

**Figure 7:**
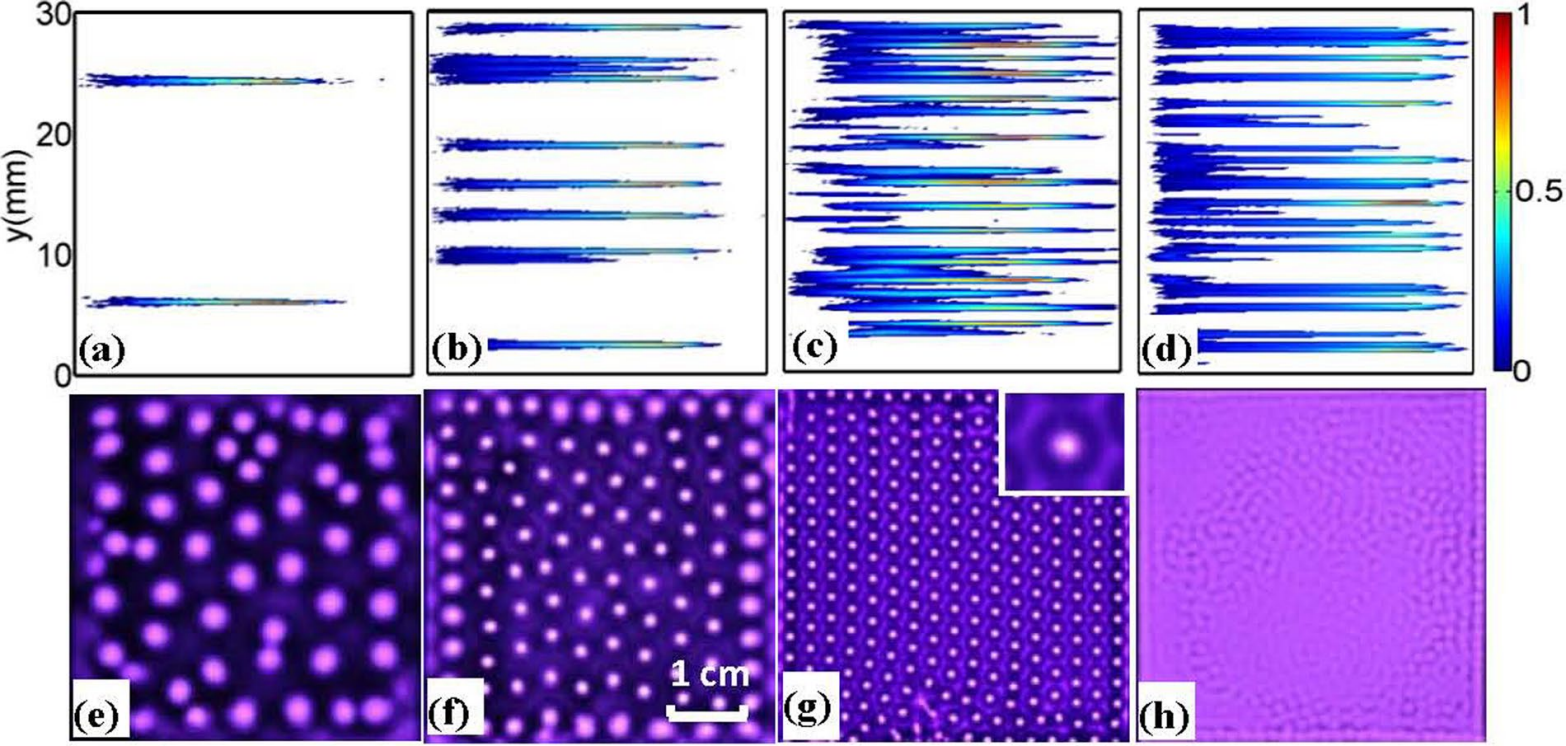
Transition of different patterns with voltage increasing. The top panel (a–d) displays the electron density distributions of the discharge in simulation, with the voltages U = 600 V, 870 V, 1200 V, 2500 V, respectively. Roughly, they are equivalent to the luminous filaments as usually observed in experiments. For simplicity, a normalized color bar is used here with the units: (**a**) 1.5 × 10^18^ m^−3^, (**b**) 8.0 × 10^18^ m^−3^, (**c**) 2.0 × 10^19^ m^−3^, (**d**) 1.1 × 10^20^ m^−3^. The bottom panel gives the experimental results: (**e**) Filaments with random arrangement, U = 1.6 kV; (**f**) Quasi-hexagon pattern, U = 1.8 kV; (**g**) Honeycomb superlattice, U = 2.2 kV, where one unit cell of the structure is magnified in the inset at the top-right corner; (**h**) Disordered state U = 3.6 kV. Experimental parameters: discharge gap *d* = 1.4 mm, voltage frequency *f* = 56 kHz, and exposure time *t* = 25 ms. The size of the discharge area is 45 × 45 mm. The experiment is performed in a mixture of He (80%) and air (20%) with gas pressure *p* = 200 Torr.

Figure 7 presents the evolution of filamentary structures as a function of the applied voltage. It can be seen in the simulation that both of the number of the filaments and the discharge intensity are remarkably increased with an increase of the applied voltage (Fig. 7(a–d)). The side discharges and the disordered discharges emerge when the voltage is sufficiently high (Fig. 7(c–d)). In good accordance of these simulation results, the discharge undergoes a transition from the random spots — quasihexagonal pattern—honeycomb superlattice—disordered state, in experiment with increasing of the applied voltage (Fig. 7(e–h)). The honeycomb superlattice is concentrated on here, in which the bright isolated spots arrange in a hexagon lattice and each spot is surrounded by a luminous honeycomb framework. It is an organizational structure with highly regularity, which naturally makes us to associate it with the periodic crystals in condensed matter fields. More importantly, it can be considered as a novel plasma photonic crystal to control the propagation of the electromagnetic waves, which has great potentials for engineering applications in optics and photonics fields^26–28^.

**Figure 8:**
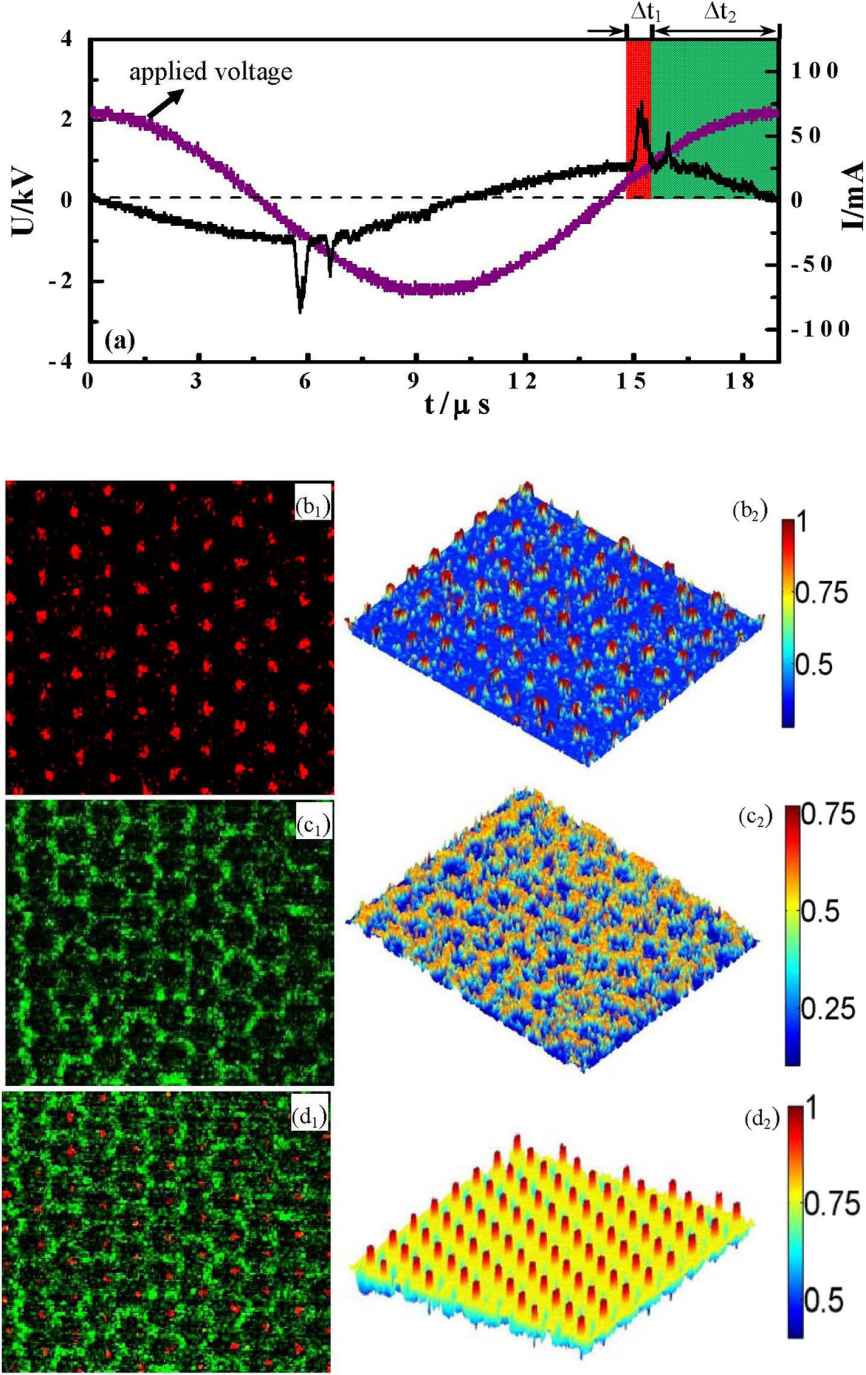
Spatial-temporal resolved measurements of the honeycomb superlattice pattern by using fast camera diagnostics. (**a**) Waveforms of the voltage and current when the honeycomb superlattice is generated. (**b**_**1**_) Snapshot of the primary hexagon lattice corresponding to the first current pulse as indicated with Δt_1_. The exposure time is 100 ns and loop 50 times. (**b**_**2**_) Normalized 3D luminance distribution of the hexagon lattice. (**c**_**1**_) Snapshot of the honeycomb framework corresponding to the second current pulse, as indicated with Δt_2_. Due to the weak intensity of the framework, the exposure time is selected to be 600 ns and loop 100 times.(**c**_**2**_) 3D Luminance distribution of the honeycomb framework. (**d**_**1**_) Overlap of the pictures (**b**_**1**_+**c**_**1**_); (**d**_**2**_) 3D luminance distribution of the honeycomb superlattice pattern that can be seen by our naked eyes, as indicated in Fig. 7(g).

By using a high speed camera, the time-resolved measurements of the honeycomb superlattice have been explored. The observation of two current pluses as indicated by the red and green areas in Fig. 8(a) suggests that the discharge takes place two times in each half cycle of the applied voltage. During the first pulse, the isolated filaments emerge and arrange in a hexagonal lattice (Fig. 8(b)). A honeycomb-like arrangement is generated during the second current pulse, which corresponds to the side discharge of the primary hexagon. It forms in the spacing of the original filaments, which is very weak and has low luminance. The light intensity of the hexagon lattice is about 1.3 times of the honeycomb framework. The honeycomb superlattice results from an interleaving of the transient hexagon lattice and the honeycomb framework. Our experimental observations are qualitatively in good agreement with the simulation results.

In summary, we present both numerically and experimentally the evolution of side discharges in a filamentary dielectric barrier discharge. Our PIC-MCC simulations show that the side discharges results from a joint action of both the transverse plasma diffusion and the ion induced secondary electron emission beyond the inhibition zone. In experiment, a novel honeycomb superlattice pattern is observed in a DBD system with two water electrodes. The existence of side discharges in the honeycomb superlattice is verified by using a high speed camera. Experimental observations and numerical simulation are in good agreement. Our results provide new insight on the underlying physics governing the discharge and explain many dynamical aspects of DBD filaments.
